# Supplementary material for: The concurrent validity and reliability of the Leg Motion system for measuring ankle dorsiflexion range of motion in older adults
Source: PeerJ. 2017 Jan 3;5:e2820. doi: 10.7717/peerj.2820 (PMC5214953; doi:10.7717/peerj.2820)
Supplement: Data S1 [file peerj-05-2820-s001.pdf]

				[illegible]
**Nombre**	**Height**	**Weight**	**BMI**	Right
1	1,79	90	28,089011	6.0
2	1,58	49	19,6282647	1.9
3	1,62	51	19,4330133	3.6
4	1,77	62	19,789971	4.4
5	1,73	80	26,7299275	7.8
6	1,57	69	27,993022	1.5
7	1,45	66	31,391201	10.0
8	1,8	85	26,2345679	3.5
9	1,61	61	23,5330427	8.5
10	1,57	49	19,8791026	1.5
11	1,65	66	24,2424242	2.6
12	1,73	82	27,3981757	7.5
13	1,59	63	24,9199003	1.5
14	1,66	55	19,9593555	4.7
15	1,53	51	21,7864924	1.5
16	1,62	52	19,8140527	6.6
17	1,86	94	27,1707712	11.5
18	1,62	73	27,8158817	6.9
19	1,63	71	26,7228725	1.4
20	1,91	79	21,6551081	6.6
21	1,84	79	23,334121	9.5
22	1,63	52	19,5716813	7.7
23	1,71	62	21,2031052	1.9
24	1,8	91	28,0864198	3.4
25	1,62	66	25,1486054	2.5
26	1,67	57	20,4381656	9.3
27	1,58	62	24,8357635	3.4
28	1,66	79	28,6688924	5.5
29	1,59	55	21,7554685	10.0
30	1,72	83	28,0557058	1.7
31	1,65	60	22,0385675	1.5
32	1,63	72	27,099251	5.8
33	1,75	86	28,0816327	4.5
	1,67090909	68,2424242	24,318289	Media
	0,10229747	13,4722639	3,50310418	DT

Leg Motion			Tape Measure			
1	2		1		2	
Left	Right	Left	Right	Left	Right	Left
5.5	5.0	6.8	5.0	6.0	4.6	5.5
4.0	1.5	3.2	2.2	4.5	2.4	3.6
4.6	3.0	4.5	3.4	5.0	3.6	5.0
5.7	5.0	5.2	4.9	4.8	4.6	5.9
6.5	6.5	7.2	8.5	6.9	7.5	6.0
1.4	2.2	1.7	1.8	1.5	1.4	1.5
7.8	9.3	7.0	10.0	6.5	10.0	7.4
2.5	4.4	3.0	4.5	2.0	3.9	2.9
7.6	7.8	7.0	7.5	7.1	8.0	7.5
2.0	2.0	2.0	1.8	2.6	2.0	2.3
4.0	x	x	3.0	3.5	x	x
9.0	6.8	9.2	6.5	8.9	6.7	9.0
3.0	2.0	3.0	1.5	3.0	1.5	3.2
7.5	5.0	7.5	4.5	6.8	4.5	7.4
1.8	x	x	2.5	2.1	x	x
5.3	7.0	5.8	6.2	5.8	5.9	6.3
12.5	12.2	12.5	12.0	11.0	12.5	12.5
5.0	6.0	5.4	6.2	5.3	7.5	5.0
1.1	1.5	1.2	1.6	1.3	1.5	1.3
7.5	5.5	7.9	5.8	8.0	6.0	8.2
10.2	10.5	9.5	9.0	11.0	9.6	10.5
8.2	x	x	8.1	8.6	x	x
2.5	2.0	3.0	2.0	3.1	2.2	2.1
4.5	2.4	4.0	2.5	3.9	2.2	4.0
1.5	2.5	2.4	2.5	1.9	3.5	2.5
7.5	9.3	6.8	9.2	6.8	9.5	6.7
2.5	4.0	2.5	4.0	2.0	5.1	3.0
4.0	5.5	4.5	6.2	4.5	5.5	4.5
9.5	10.5	8.7	11.5	9.4	11.4	9.4
3.0	2.0	3.0	1.5	3.0	1.4	3.0
1.7	1.5	2.5	1.8	2.5	1.9	3.3
7.8	4.4	7.0	5.0	7.0	5.5	7.2
2.5	4.9	2.0	4.5	1.5	4.7	1.5

Goniometer				Inclinometer		
1		2		1		2
Right	Left	Right	Left	Right	Left	Right
47	48	42	42	54	46	43
49	51	46	46	52	44	44
42	39	46	40	49	43	50
45	47	42	45	46	42	49
41	47	41	45	47	40	49
48	49	47	53	38	48	45
39	47	42	51	48	44	46
49	52	53	47	47	44	45
44	42	40	37	35	37	37
57	50	54	47	46	50	52
49	44	x	x	44	42	x
46	43	44	44	55	49	50
54	52	50	49	55	46	51
44	41	42	46	40	48	43
46	48	x	x	48	55	x
42	47	46	49	47	40	40
38	32	43	31	46	39	49
42	46	44	50	45	45	42
57	59	50	52	44	59	49
41	36	48	42	40	37	42
37	35	32	42	45	41	41
42	39	x	x	45	43	x
49	42	46	48	48	47	45
44	48	40	52	49	39	49
51	44	45	46	44	51	48
44	49	48	42	44	42	42
48	51	42	46	52	43	44
47	45	39	42	43	52	46
42	46	44	49	50	49	48
46	44	42	44	47	48	48
49	47	55	52	41	55	46
44	42	42	47	50	48	45
50	44	44	41	49	42	48

[illegible]
Left
44
45
49
45
46
53
44
52
39
45
x
54
51
41
x
42
46
51
57
39
39
x
41
44
47
40
46
53
51
45
58
49
41
